# Ethnic differences in ovulatory function in nulliparous women

**DOI:** 10.1038/sj.bjc.6600098

**Published:** 2002-02-01

**Authors:** C A Haiman, M C Pike, L Bernstein, S V Jaque, F Z Stanczyk, A Afghani, R K Peters, P Wan, L Shames

**Affiliations:** Department of Preventive Medicine, USC/Norris Comprehensive Cancer Center, Los Angeles, California, USA; Department of Biokinesiology & Physical Therapy, Keck School of Medicine, University of Southern California, Los Angeles, California, USA; Department of Obstetrics and Gynecology, Keck School of Medicine, University of Southern California, Los Angeles, California, USA

**Keywords:** oestrogens, ethnicity, ovulation

## Abstract

African-American women have a long-standing approximately 20% higher breast cancer incidence rate than USA White women under age 40 while rates among Latinas are lower than those of Whites. The reasons for this are not clear, however they may be due to ethnic differences in circulating oestradiol and progesterone levels. In a cross-sectional study, we investigated whether anovulation frequency and circulating serum oestradiol and/or progesterone levels vary among normally cycling nulliparous African-American (*n*=60), Latina (*n*=112) and non-Latina White (*n*=69) women. Blood and urine specimens were collected over two menstrual cycles among healthy 17- to 34-year-old women. Frequency of anovulation was greater among White women (nine out of 63, 14.3%) than African-American women (four out of 56, 7.1%) or Latina women (seven out of 102, 6.9%), although these differences were not statistically significant. African-American women had 9.9% (*P*=0.26) higher follicular phase oestradiol concentrations than Latina women and 17.4% (*P*=0.13) higher levels than White women. African-American women also had considerably higher levels of luteal phase oestradiol (*vs* Latinas, +9.4%, *P*=0.14; *vs* Whites, +25.3%, *P*=0.003) and progesterone (*vs* Latinas, +15.4%, *P*=0.07; *vs* Whites, +36.4%, *P*=0.002). Latina women were also observed to have higher follicular oestradiol, and luteal oestradiol and progesterone levels than White women (follicular oestradiol: +6.8%, *P*=0.48; luteal oestradiol: +14.6%, *P*=0.04; luteal progesterone: +18.2%, *P*=0.06). These results suggest that exposure to endogenous steroid hormones may be greater for young African-American and Latina women than for Whites.

*British Journal of Cancer* (2002) **86**, 367–371. DOI: 10.1038/sj/bjc/6600098 www.bjcancer.com

© 2002 The Cancer Research Campaign

## 

African-American women have a long-standing approximately 20% higher breast cancer incidence rate than USA White women under age 40, while rates among Latinas are lower than those of Whites (Gray *et al*, 1980; Parkin *et al*, 1997; SEER Program, 2001). The reasons for this are not clear, however they may be due to ethnic differences in circulating oestradiol (E_2_) and progesterone (P_4_) levels. Oestradiol is an established breast-cell mitogen, and considerable epidemiological and experimental evidence indicates that women with higher circulating levels are at greater risk of developing breast cancer (Bernstein and Ross, 1993; Pike *et al*, 1993; Thomas *et al*, 1997; Hankinson *et al*, 1998). Considerable evidence also exists indicating that progestins play an important role in breast carcinogenesis. Recent epidemiological studies have shown that postmenopausal hormone replacement therapy in the form of estrogen–progestin replacement therapy (EPRT) increases breast cancer risk to a much greater extent than estrogen (alone) replacement therapy (ERT; Magnusson *et al*, 1999; Ross *et al*, 2000; Schairer *et al*, 2000). These results are supported by the finding that mammographic densities are increased much more by EPRT than ERT (Greendale *et al*, 1999), and so is breast-cell proliferation (Hofseth *et al*, 1999).

In the present study, we evaluated differences in ovarian function in African-American, Latina and non-Latina White young women in Los Angeles who were recruited as part of a cross-sectional investigation of the frequency of anovulation and of circulating serum E_2_ and P_4_ levels among ‘normally cycling’ nulliparous women.

## SUBJECTS AND METHODS

We collected blood and urine specimens and questionnaire data from 241 healthy female college students, who were not athletes. Most subjects were studied over two menstrual cycles.

### Subjects

Study subjects had to meet the following criteria: 18 to 35 years of age; nulliparous; free of underlying conditions or medication use (within the previous 6 months) that may interfere with ovulation and/or hormone levels (specifically no use of cimetidine, thyroid supplements, or ω-3 fatty acid supplements; no general anaesthesia); no hormonal contraceptive use over the previous 6 months and no planned use of a hormonal contraceptive over the course of the two menstrual cycles being studied; and with self-reported ‘regular cycles’. Volunteers were recruited at colleges within the Los Angeles area: University of Southern California, California State University at Los Angeles, Santa Monica College, Los Angeles Community College, Los Angeles Trade Tech, and West Los Angeles College. Volunteers were sought by posting fliers around campuses, by placing advertisements in college newspapers, and by making brief presentations to students attending classes in the science and health science fields. Eligible volunteers were asked to notify us of the first day of their next menstrual cycle. At that time, subjects were scheduled for an appointment to complete a self-administered questionnaire, to receive urine-collection bottles, and to receive information concerning the conduct of the study and their scheduled blood-draw appointments. The study protocol was approved by the Institutional Review Board of the Keck School of Medicine of the University of Southern California.

### Questionnaire data collection

The self-administered questionnaire included basic demographic questions (age, ethnicity), age at menarche, use of tobacco and alcohol, and a section on exercise habits.

Our main measure of exercise was the ‘College Alumnae Questionnaire‘ (CAQ) developed by Paffenbarger *et al* (1993), which quantifies exercise by type, intensity and duration. When this questionnaire is used in conjunction with the ‘Compendium of Physical Activities – Physical Activity Index’ (CPA–PAI), it allows for the computation of metabolic activity in MET-minutes, i.e., duration of activity multiplied by the ratio of the metabolic rate for the specific activity divided by the resting metabolic rate (Ainsworth *et al*, 1993a). This has been validated in both women and men, and has been used extensively in studies of the association of exercise activity and heart disease rates, longevity, cardiorespiratory fitness and energy balance (Ainsworth *et al*, 1993b). The CPA–PAI allows for a detailed description of the form and intensity of most exercise activities. To reduce variation in coding of exercise activities, one of us (A Afghani) coded all questionnaires.

### Sample collection

During the first menstrual cycle (‘blood cycle’), subjects were asked to provide from two to five blood samples (depending on the length of the cycle). Counting the first day of menstrual bleeding as cycle day 1, blood was drawn on cycle days 11 (±1) and 22 (±1), and subsequently at weekly intervals on days 29 (±1), 36 (±1) and 42 (±1) until menses occurred. We allowed a ±1 day variation in collection date to avoid having to collect samples on weekends and to allow for unavoidable conflicts. Subjects reported to a local laboratory between 07:00 and 09:30 h in a fasting state and having refrained from exercise that morning prior to the blood draw. Serum was separated, aliquoted and stored at −86°C at our central laboratory. During a second menstrual cycle (‘urine cycle’), first morning urine samples were collected on cycle day 10, and subsequently every 4th day until the first day of the next cycle. Participants were asked to place urine samples in their home refrigerators and to bring urine specimens in a freezer lunch bag with frozen blue ice (supplied in the kit) to a drop-off refrigerator (4°C) at their respective colleges for pick-up. Samples were then catalogued, aliquoted and stored at −86°C at our central laboratory.

### Hormone assays

Serum E_2_, P_4_, and urinary pregnanediol glucuronide (Pdiol-3G) were measured in the laboratory of Dr Stanczyk. Laboratory technicians were blinded to the ethnicity and other details of study subjects. Serum E_2_ was measured following extraction with ethyl acetate : hexane, using an iodinated radioimmunoassay (RIA) kit from Pantex (Santa Monica, California, USA) as described previously (Stanczyk *et al*, 1988). The intra-assay coefficient of variation (CV) is 10.9% and the inter-assay CV is 9.1%. Serum P_4_ was measured by RIA after extraction with hexane as described previously (Scott *et al*, 1978). The intra-assay CV is 6.2% and the inter-assay CV is 8.7%. Urinary Pdiol-3G was measured by direct RIA as described previously (Stanczyk *et al*, 1980). The intra-assay CV is 3.1% and the inter-assay CV is 10.9%. Urinary Pdiol-3G concentrations are expressed per urinary concentration of creatinine, which was measured colourimetrically.

### Determination of ovulation

We classified each blood cycle as ovulatory, anovulatory or indeterminate based on the availability (and value) of a serum P_4_ on days −3 to −11 (i.e., the availability of a serum P_4_ between 3 and 11 days before the onset of the next menses). A serum P_4_ >3.0 ng ml^−1^ was considered as indicative of ovulation and only ovulatory cycles were used in the analyses of serum hormone levels. For these analyses, the luteal phase sample that was closest to the onset of the next menses within the −3 to −11 range was used for analyses of luteal phase E_2_ and P_4_ levels.

We classified each urine cycle as ovulatory, anovulatory or indeterminate based on the availability (and value) of urinary Pdiol-3G on days −3 to −11 (i.e., the availability of a Pdiol-3G between 3 and 11 days before the onset of the next menses); only one value per subject during the luteal phase was used (the one closest to the onset of the next menses within the acceptable range). A Pdiol-3G level >1.25 μg mg^−1^ was considered indicative of ovulation (see Discussion).

### Statistical methods

Logistic regression models were used for the analysis of anovulation frequency, with adjustment for BMI (body-mass index; quartiles), exercise (MET-mins/day; tertiles), smoking (never, past, current), alcohol (never, past, current), age at menarche (⩽11, 12, 13, 14+ years), gynaecologic age (current age – age at menarche; ⩽8, 8+ years), cycle length (⩽26, 27–28, 29–30, 31–32, 33+ days) and days to start of next menstrual cycle (3–5, 6–8, 9–11 days). Geometric mean serum E_2_ and P_4_ values were compared across the three ethnic groups using multiple regression methods, with adjustment for the variables described above for the luteal phase analysis; and for the follicular phase (day 11) E_2_ analysis adjustment was made for the variables described above but the days to start of next menstrual cycle groups were changed to ⩽17, 18–19, 20–21, and 22+ days.

## RESULTS

Two hundred and forty-one eligible women between the ages of 17 and 34 provided specimens for the study; of these 60 were African Americans, 112 were Latinas (Mexicans or other Central American Hispanics) and 69 were non-Latina Whites (herein referred to as Whites). Ovulatory status during the blood cycle could be determined for 221 of the 241 women, i.e., we had a P_4_ value for 221 of the 241 women in the −3 to −11 day range. During the urine cycle, we restricted the analysis to women whose ovulatory status could be determined during the blood cycle; ovulatory status was determined for 152 women.

Descriptive characteristics by ethnic group are presented in Table 1

**Table 1:**
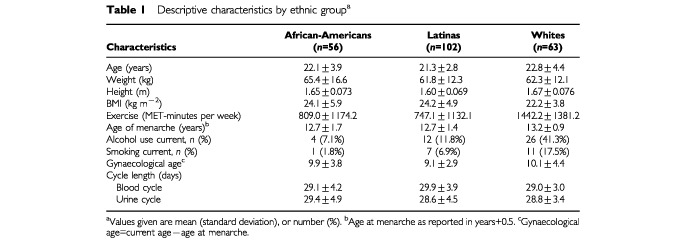
Descriptive characteristics by ethnic group^a^

. The White women began menstruating at a later age than African-American and the Latina women (*P*=0.038). The White women also spent more time exercising (*P*=0.001) and were more likely to be a current drinker of alcohol or a smoker than the African-American or Latina women. The mean BMI for African-Americans and Latinas was significantly greater than that of Whites (*P*=0.032). Gynaecologic age and cycle lengths were similar in the different ethnic groups.

The proportion of women classified as anovular differed by ethnic group (Table 2

**Table 2:**
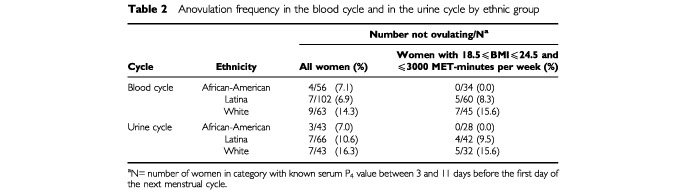
Anovulation frequency in the blood cycle and in the urine cycle by ethnic group

), however, the differences were not statistically significant. During the blood cycle, 7.1% of the African-American, 6.9% of the Latina and 14.3% of the White women were classified as anovular. A similar racial/ethnic pattern was observed after adjustment for possible predictors of ovulation status (see Statistical methods section; data not shown). A similar pattern was also seen during the urine cycle (Table 2).

The African-American and Latina women categorized as anovular during the blood cycle were heavier and reported fewer MET-minutes per week of exercise than ovular women in their ethnic groups. The BMIs of the anovular African-American women (*n*=4; 16.9, 33.4, 40.8 and 49.8 kg m^−2^) were on the extremes of the BMI distribution for African-Americans in this study. The BMIs for the anovular Whites were between 20.5 and 25.2 kg m^−2^. The greater frequency of anovulation of the White women remained when the analysis was restricted to women with BMIs within the ‘normal healthy’ range (18.5–24.9 kg m^−2^) and who exercised in the moderate range (⩽3000 MET-minutes per week; Table 2); in this subgroup of women, none of the African-American, 8.3% of the Latina and 15.6% of the White women were anovular.

Geometric mean follicular serum E_2_ and luteal phase serum E_2_ and P_4_ levels among the ovulating women are shown in Table 3

**Table 3:**
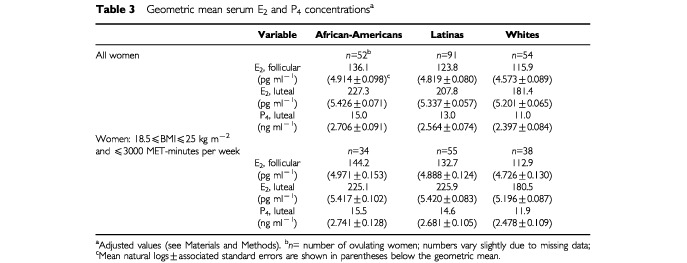
Geometric mean serum E_2_ and P_4_ concentrations^a^

. In adjusted analyses, the African-Americans had 9.9% (*P*=0.26) higher levels of follicular E_2_ than the Latina women and 17.4% (*P*=0.13) higher levels than the White women. The African-American women also had substantially higher mean luteal phase levels of E_2_ (*vs* Latinas, +9.4%, *P*=0.14; *vs* Whites, +25.3%, *P*=0.003) and P_4_ (*vs* Latinas, +15.4%, *P*=0.07; *vs* Whites, +36.4%, *P*=0.002). Follicular E_2_, and luteal E_2_ and P_4_ levels were higher for Latinas than Whites (follicular E_2_: +6.8%, *P*=0.48; luteal E_2_: +14.6%, *P*=0.04; luteal P_4_: +18.2%, *P*=0.06). The racial/ethnic differences in serum hormone levels were also observed among women with BMIs within the normal weight range (18.5–24.9 kg m^−2^) who exercised in the moderate range (⩽3000 MET-minutes per week; Table 3).

## DISCUSSION

Little is known regarding ethnic differences in ovarian function. Using National USA Survey data, MacMahon (1973) first described African-Americans as having an earlier age at menarche than Whites and this was also found in a recent study conducted among a cohort of schoolgirls in Los Angeles (Koprowski *et al*, 1999). Koprowski *et al* (1999) also found the White girls to have a later age at menarche than Latina girls, but there was no difference between the African-Americans and the Latinas. The White women in our study also began menstruating at a significantly later age. We now provide evidence that White women have a greater frequency of anovular menstrual cycles than African-American and Latina women. Although anovulation among African-American women could be explained by extremes in BMI, and Whites exercised more than African-Americans and Latinas, extremes in BMI or greater exercise among Whites could not account for the observed racial/ethnic differences in anovulation frequency. When limiting the analysis to women who did not exercise vigorously (⩽3000 MET-minutes per week) and had BMIs within the ‘normal-healthy’ range (18.5–24.9 kg m^−2^), Whites were still observed to have a greater frequency of anovular menstrual cycles than African-Americans.

The ethnic differences in anovulation frequency that we observed during the blood cycle remained when the acceptable window of days to the onset of next menses was further restricted from −4 to −11 or −5 to −10, and also when the P_4_ level cut-point used to define an ovulatory cycle was increased to 4.0 or 5.0 ng ml^−1^. The −3 to −11 range of informative days was based on the results presented by Israel *et al* (1972) and inspection of our results when we had multiple P_4_ values for participants. Israel *et al* (1972) suggest that to define whether ovulation has occurred on the basis of a P_4_ value, the specimen should be drawn from 4 to 11 days before the onset of the next menses rather than from 3 to 11 days; however, we found no inconsistencies when we used the slightly wider time frame. During the urine cycle, a Pdiol-3G to creatinine ratio greater than 1.25 μg mg^−1^ was initially selected to define ovulatory status because this cut-point produced a comparable overall frequency (i.e., over all groups combined) of anovulation to that observed in the blood cycle. As with the serum determination of ovulation, the between ethnic group comparisons were not sensitive to the specified permissible window, but they were to the Pdiol-3G/creatinine cut-point. Ethnic differences were no longer apparent when a Pdiol-3G value >1.75 ng mg^−1^ creatinine was used to define ovulatory status; at this cut-point approximately 20% of each ethnic group were classified as anovular. This high percentage is not compatible with the results from the serum P_4_ level determination, and we believe the results at the 1.25 μg mg^−1^ cut-point more likely represent the true anovulation incidence. However, the literature on the ‘correct’ cut-point is sparse, and before future studies use Pdiol-3G to determine ovulatory status, studies need to be performed to establish whether the critical Pdiol-3G/creatinine level is uniform across ethnic groups, as we have assumed here.

Differences in premenopausal steroid hormone levels between African-American and White women have been reported previously, however, data are limited and comparisons with Latinas have not been investigated. In a diet-intervention study, Woods *et al* (1996) observed at baseline that African-American women (*n*=21) had a 55% higher follicular-phase E_2_ level than Whites (*n*=68; *P*⩽0.001). In a study of pregnant women, Henderson *et al* (1998) also observed African-American women to have higher steroid hormone levels than White women during the first trimester of pregnancy. Our results support these findings, as we observed African-American women to have higher levels of both follicular E_2_ and luteal E_2_ and P_4_ than Latina and White women.

Our data suggest that the higher breast cancer incidence of African-American women under 40 years of age may be due in part to ethnic differences in ovarian function; we observed African-American women to have an earlier age at menarche, a greater frequency of ovular cycles, and, among ovulating women, higher circulating levels of E_2_ and P_4_. We also provide evidence that Latina women have an earlier age at menarche and higher luteal E_2_ and P_4_ hormone levels than White women. The Latina women in this study were identified from colleges in the Los Angeles area and do not include Latinas of low SES. Although overall breast cancer rates in young Latina women in Los Angeles are some 25% lower than the rates in Whites, the rates in middle class Latinas are only 12% lower than in Whites (unpublished data from the Los Angeles County Cancer Surveillance Program, part of the SEER network of registries). Based on these findings, one may predict that breast cancer rates among young Latina women may soon rise to at least the high breast cancer rates observed in White women.

In summary, this paper supports the work of others in demonstrating ethnic differences in ovarian function and further adds to the sparse data available in the Latina population. In order to gain a more comprehensive and thorough understanding of the complexities and nuances associated with this extremely dynamic system, daily measurements of gonadotropins and steroid hormones collected over multiple menstrual cycles among a large sample of women are needed. Inter-individual and inter-ethnic differences in ovarian function are most likely due to the collective effects of nutrition, energy expenditure, caloric intake, body size and genetic variation. In addition to monitoring physical activity, future studies will also need to collect comprehensive dietary records to examine the relationship between energy balance and ovulatory function. Research efforts should also be aimed at identifying the relevant genes involved in regulating gonadotropin and steroid hormone production, and investigating whether functional polymorphisms in these genes lead to variation in ovarian function.
